# Identifying clinical subtypes in sepsis-survivors with different one-year outcomes: a secondary latent class analysis of the FROG-ICU cohort

**DOI:** 10.1186/s13054-022-03972-8

**Published:** 2022-04-21

**Authors:** Sabri Soussi, Divya Sharma, Peter Jüni, Gerald Lebovic, Laurent Brochard, John C. Marshall, Patrick R. Lawler, Margaret Herridge, Niall Ferguson, Lorenzo Del Sorbo, Elodie Feliot, Alexandre Mebazaa, Erica Acton, Jason N. Kennedy, Wei Xu, Etienne Gayat, Claudia C. Dos Santos, the FROG-ICU, Sabri Soussi, Alexandre Mebazaa, Etienne Gayat, CCCTBG trans-trial group study for InFACT - the International Forum for Acute Care Trialists, Sabri Soussi, Laurent Brochard, John C. Marshall, Margaret Herridge, Claudia C. Dos Santos

**Affiliations:** 1 Interdepartmental Division of Critical Care, Faculty of Medicine, St Michael’s Hospital, Keenan Research Centre for Biomedical Science and Institute of Medical Sciences, University of Toronto, 209 Victoria St 7th Floor, Toronto, ON M5B 1T8 Canada; 2 Department of Biostatistics, Princess Margaret Cancer Centre, University of Toronto, Toronto, ON Canada; 3 Applied Health Research Centre, Li Ka Shing Knowledge Institute of St Michael’s Hospital, Toronto, ON M5B 1W8 Canada; 4 Department of Medicine and Institute of Health Policy, Management and Evaluation, University of Toronto, Toronto, ON Canada; 5 Peter Munk Cardiac Centre, University Health Network, and Heart and Stroke Richard Lewar Centre of Excellence in Cardiovascular Research, University of Toronto, Toronto, ON Canada; 6 Department of Medicine, Interdepartmental Division of Critical Care Medicine, Toronto General Research Institute, Institute of Medical Science, University Health Network, University of Toronto, Toronto, ON Canada; 7 Department of Anesthesiology, Critical Care, Lariboisière - Saint-Louis Hospitals, DMU Parabol, AP–HP Nord; Inserm UMR-S 942, Cardiovascular Markers in Stress Conditions (MASCOT), University of Paris, Paris, France; 8 Department of Critical Care Medicine, School of Medicine, University of Pittsburgh, Pittsburgh, PA USA

**Keywords:** Sepsis, Post-intensive care syndrome (PICS), Biomarkers, Latent profile analysis, Mixture modeling, Prognostic enrichment, Personalized medicine

## Abstract

**Background:**

Late mortality risk in sepsis-survivors persists for years with high readmission rates and low quality of life. The present study seeks to link the clinical sepsis-survivors heterogeneity with distinct biological profiles at ICU discharge and late adverse events using an unsupervised analysis.

**Methods:**

In the original FROG-ICU prospective, observational, multicenter study, intensive care unit (ICU) patients with sepsis on admission (Sepsis-3) were identified (*N* = 655). Among them, 467 were discharged alive from the ICU and included in the current study. Latent class analysis was applied to identify distinct sepsis-survivors clinical classes using readily available data at ICU discharge. The primary endpoint was one-year mortality after ICU discharge.

**Results:**

At ICU discharge, two distinct subtypes were identified (A and B) using 15 readily available clinical and biological variables. Patients assigned to subtype B (48% of the studied population) had more impaired cardiovascular and kidney functions, hematological disorders and inflammation at ICU discharge than subtype A. Sepsis-survivors in subtype B had significantly higher one-year mortality compared to subtype A (respectively, 34% vs 16%, *p* < 0.001). When adjusted for standard long-term risk factors (e.g., age, comorbidities, severity of illness, renal function and duration of ICU stay), subtype B was independently associated with increased one-year mortality (adjusted hazard ratio (HR) = 1.74 (95% CI 1.16–2.60); *p* = 0.006).

**Conclusions:**

A subtype with sustained organ failure and inflammation at ICU discharge can be identified from routine clinical and laboratory data and is independently associated with poor long-term outcome in sepsis-survivors.

*Trial registration* NCT01367093; https://clinicaltrials.gov/ct2/show/NCT01367093.

**Graphical Abstract:**

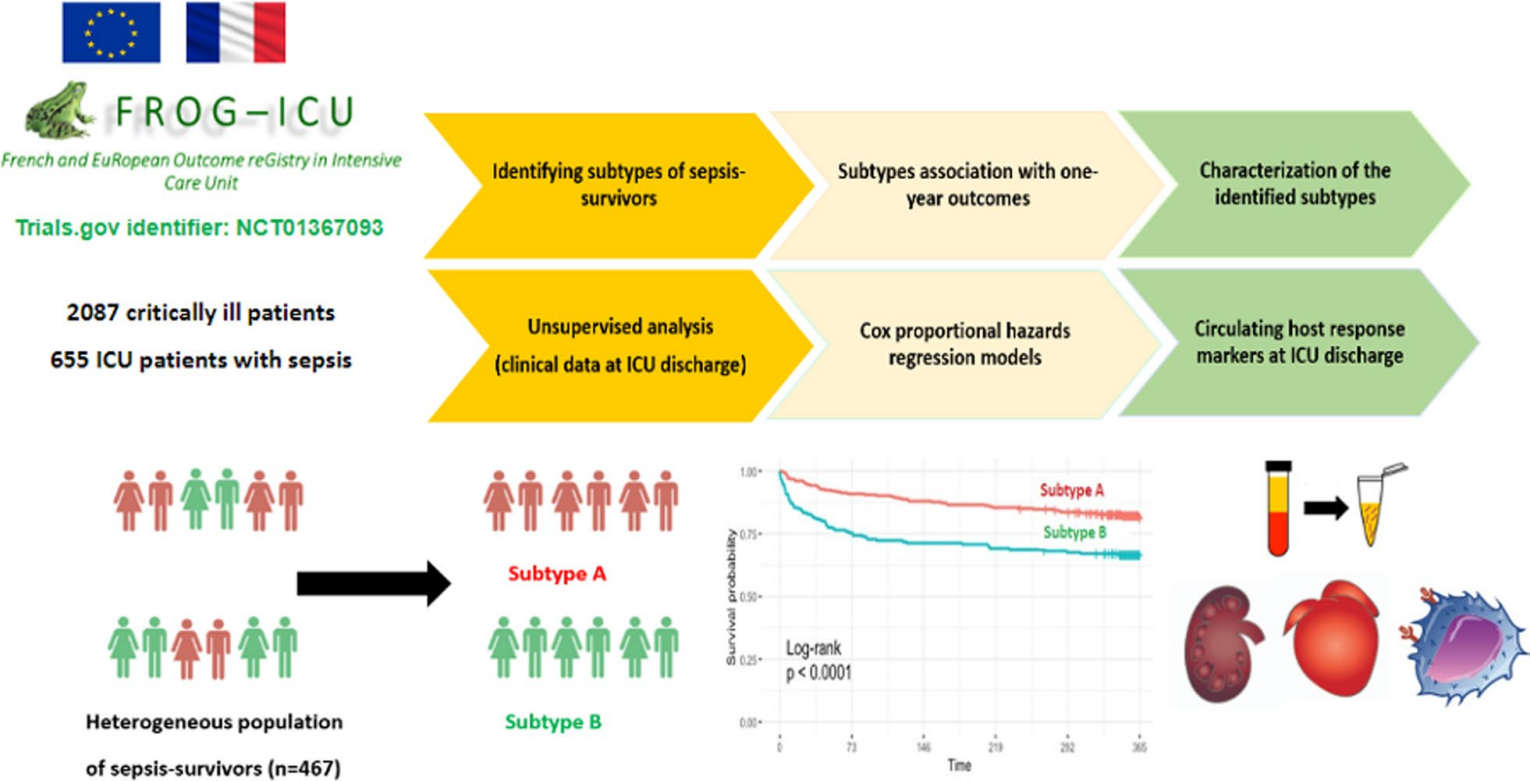

**Supplementary Information:**

The online version contains supplementary material available at 10.1186/s13054-022-03972-8.

## Introduction

Sepsis is a life-threatening dysregulated response to infection leading to multiorgan dysfunction [1]. Advances in critical care medicine have decreased early hospital mortality, thus increasing the number of septic patients who survive and are discharged from the hospital with sequelae of critical illness [2, 3]. However, late mortality risk after sepsis persists for years with high readmission rates and low quality of life [4, 5]. Efforts to implement preventive interventions are limited by an incomplete understanding of the relevant intermediary causal mechanisms of post-sepsis syndrome [6, 7].

The present study seeks to identify ‘hidden’ subtypes of sepsis-survivors using an unsupervised approach (i.e., clustering regardless of outcome) at intensive care unit (ICU) discharge with simple clinical and laboratory parameters. This approach has been used in the acute phase of sepsis mostly using complex inflammatory markers for prognostic and predictive enrichment [8–10] but only scarce data exist in the study of sepsis-survivors and their long-term adverse events [4].

A subtype-guided approach at ICU discharge may enable a better understanding of sepsis-survivors trajectory in the ICU (e.g., latent profiles of persistent organ dysfunction, immunosuppression and inflammation after stabilization) [11]. This may allow prognostic and therapeutic enrichment (i.e., selection of patients with a greater likelihood of having an endpoint or to respond to the drug treatment independently from initial severity of illness) to support future randomized controlled trials of therapies or prevention strategies in sepsis-survivors [12].

We analyzed existing clinical and molecular data collected from the French and European Outcome Registry in Intensive Care Units (FROG-ICU) prospective observational cohort study [13, 14]. The primary goal of the study was to determine whether readily available clinical and biological data at ICU discharge could identify distinct clinical classes (labeled as subtypes) in sepsis-survivors using an unsupervised approach (i.e., latent class analysis). The secondary goals were to determine whether identified classes are associated with distinct organ dysfunction and host response circulating markers and different long-term outcomes in ICU survivors.

## Methods

A secondary analysis of the FROG-ICU dataset was performed (trials.gov identifier: NCT01367093, registered June 6, 2011). The FROG-ICU study was a prospective, observational, international cohort study with a biobank (plasma and urine), including adult critically ill patients, designed to assess the incidence and to identify risk factors for mortality during the first year following discharge from the ICU. Study design details are published [13, 14]. The study was conducted in France and Belgium in accordance with Good Clinical Practice (Declaration of Helsinki 2002) and Ethical Committee approvals (Comité de Protection des Personnes—Ile de France IV, IRB n°00003835 and Commission d’éthique biomédicale hospitalo-facultaire de l’hôpital de Louvain, IRB n° B403201213352). Current reanalysis of the FROG-ICU cohort was performed in compliance with Unity Health Toronto (Toronto, Ontario, Canada) Research Ethics Board (n° 19-138). Written consent was waived; all patients and/or next of kin were informed and verbal consent was documented in the patients’ medical records.

Reporting of this study was in accordance with the Strengthening the Reporting of Observational Studies in Epidemiology (STROBE) statement and guidelines [15].

### Study population

In the original FROG-ICU cohort, patients were included from August 2011 to June 2013 [13, 14]. The study involved 21 ICUs in 14 university hospitals. Inclusion criteria were invasive mechanical ventilation support or treatment with vasopressors (except dopamine) for more than 24 h. Exclusion criteria were age less than 18 years, pregnancy or breastfeeding, severe head injury (initial Glasgow Coma Scale ≤ 8), brain death or a persistent vegetative state, transplantation in the past 12 months, moribund patient or no social security coverage.

In the current study, patients with sepsis or septic shock on admission (i.e., the reported main cause of admission is sepsis) or within the first 24 h after inclusion in the original FROG-ICU study (Sepsis-3 definition) [1] and who were discharged alive from the ICU were included. Among them, patients on systemic chronic immunosuppressive treatments (e.g., corticosteroids, chemotherapy) before ICU admission were excluded from the reanalysis. For included patients who had multiple admissions to ICU, only the first ICU admission was considered for reanalysis.

Sepsis-2 criteria were determined at enrollment in the whole FROG-ICU cohort. Retrospectively, we ascertained/adjudicated that all patients that met sepsis criteria on ICU admission also met Sepsis-3 criteria as per reported main cause of admission, clinical notes and clinical and biological data within the first 24 h after inclusion. Patients with documented (i.e., systemic administration of antibiotics and a positive body fluid culture specimen) or suspected infection and the presence of organ dysfunction defined as two or more Sequential Organ Failure Assessment (SOFA) points were considered as meeting the Sepsis-3 criteria [1]. When SOFA score was not available, a retrospective calculation of its different components was performed based on the available data.

### Study objectives and outcomes

The primary endpoint was all cause mortality one year after ICU discharge. Secondary outcomes were all cause mortality three and six months after ICU discharge, readmissions within the first year after ICU discharge and health-related quality of life assessed by short form-36 questionnaire (SF-36) with its physical and mental component scores (PCS and MCS) one year after ICU discharge [16–18]. At three, six and twelve months after ICU discharge, patients or their families were contacted by phone and information about vital status, readmission and quality of life was recorded. The readmissions were confirmed by reviewing the clinical notes of the readmitting hospital. For patients lost of follow-up, the vital status was checked through the national health services.

### Data collection and candidate variables for phenotyping

Clinical and biological data were recorded at inclusion and at discharge. Severity scores and Charlson age–comorbidity Index were calculated at inclusion. The Charlson age–comorbidity Index combines 19 medical conditions weighted 1–6, with age weighted 1 for every decade past 40 years [19–21]. Details of data collection have been previously reported in full [13, 14]. In addition to routine biological data, circulating markers (e.g., inflammatory, cardiovascular and renal biomarkers) were collected at inclusion and at ICU discharge and measured centrally in the original study. Details of the biomarkers and the methods used to perform the assays were previously published [13, 14, 22–24].

The list of clinical and biological variables at discharge to be included in latent class analysis (LCA) was determined a priori. Available clinical and biological data at ICU discharge were preselected as candidate variables based on the prior published literature [5, 14, 25]. Variables with more than 25% missing values were excluded from the selection process. The final selection of variables included in the LCA models (15 variables) was made by consensus among three critical care medicine experts: AM, LB and JM (Additional file 1: Table S1). The selected variables correlations are summarized in Additional file 1: Fig. S1. For each variable, the most abnormal value within the last 48 h before ICU discharge was extracted.

Thereafter, circulating markers measured at ICU discharge (details described in Additional file 1: Table S2) were compared across the identified classes (labeled as subtypes).

### Statistical analysis

Sample size calculation (*N*) to determine the impact of risk factors associated with one-year all-cause mortality was performed based on the primary endpoint from the original FROG-ICU study [13]. Missing values were handled by multiple imputation by chained equations (R-package ‘mice’) [26]. A total of 20 imputed datasets were generated. Log transformation was used for non-normal data.

LCA was secondarily used (R-package ‘depmixS4’, ‘mix’ function) [27] to identify classes of sepsis-survivors based on their clinical and routine biological data at ICU discharge. Classification was conducted independently of clinical outcome. Only variables with less than 25% missing data were considered within the LCA. After evaluating correlation, highly correlated variables using Spearman's rank-order statistics (correlation coefficient > 0.5) were excluded from the LCA and consensus k means clustering (Additional file 1: Methods) [28].

The optimal number of latent classes was decided by considering the Bayesian Information Criteria (BIC) (lower values suggest model parsimony), class interpretability (the extent to which additional classes provided clinically relevant information) and class prevalence (e.g., classes with at least 5% of the sample to improve replicability) [8, 9]. We prioritized the lowest BIC, followed by clinical significance and adequate class size [29].

The LCA model at ICU discharge was run on each of the 20 imputed datasets separately. The reported number of latent classes was the one selected in the most imputed data sets. Thereafter, final class assignment was determined by taking the majority votes of the 20 LCA models for each patient [10, 30].

To assess the reproducibility of the classes with the same selected variables, consensus k means clustering was applied using the aforesaid approach regarding missing data and highly correlated variables. The optimal number of latent classes selection is described in Additional file 1: Methods.

The associations between subtypes membership and one-year mortality and secondary outcomes were analyzed. Basic characteristics and circulating markers levels comparisons between subtypes groups were conducted at ICU discharge. Continuous variables were expressed as median (IQR) and were compared with the Mann–Whitney U test. Categorical variables were expressed as frequencies and percentages and were compared with the Fisher exact test or the Chi square test as appropriate. To examine the impact of class membership on one-year mortality, survival curves were generated by the Kaplan–Meier analyses. Multivariable survival analysis was performed using Cox proportional hazards regression models. Subtype membership at ICU discharge and one-year mortality risk factors [5, 14, 18] were included in the Cox regression models. A logistic regression model was also constructed to identify the main biomarkers measured at discharge associated with classes membership [31–33]. Details of regression models’ analysis were reported in Additional file 1: Methods. Two-sided tests were applied with *p* ≤ 0.05 considered statistically significant. All the analyses were performed using the R statistical software (https://www.r-project.org/).

## Results

**Fig. 1:**
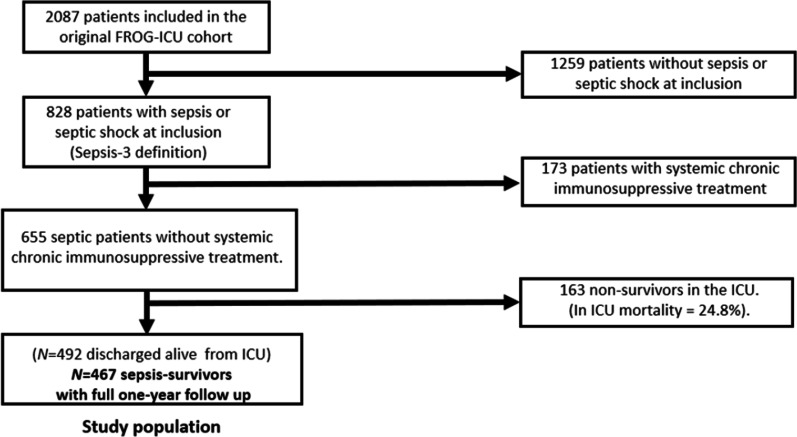
Study flowchart. Abbreviation: *ICU* intensive care unit

The study flowchart is represented in Fig. 1. Patients’ characteristics are summarized in Table 1 and Additional file 1: Table S3. Sites of infection and microbiological characteristics are summarized in Additional file 1: Table S4. One-year mortality after ICU discharge was 24.6%. Median Charlson age–comorbidity index and ICU length of stay for sepsis-survivors were 3 (2–5) and 13 (8–22) days respectively. On admission, 91.2% of sepsis-survivors required mechanical ventilation and 86.2% required vasopressors.

**Table 1:** Patient characteristics and outcomes based on subtypes at ICU discharge

	All patients (*N* = 467)	Subtype A (*N* = 244)	Subtype B (*N* = 223)	*p* value
Age, years^†^	64 (53–75)	60 (49–70)	69 (58–78)	< 0.001
Male gender	293 (62.7%)	141 (57.7%)	152 (68.1%)	0.028
BMI, kg/m^2†^	27 (23–31)	26 (22–29)	28 (24–32)	0.001
Comorbidities^†^				
Charlson age–comorbidity index	3 (2–5)	3 (1–4)	4 (3–6)	< 0.001
Diabetes mellitus, *n* (%)	98 (20.9%)	34 (13.9%)	64 (28.6%)	< 0.001
Chronic heart failure, *n* (%)	35 (7.4%)	10 (4.1%)	25 (11.2%)	0.004
Coronary artery disease, *n* (%)	40 (8.5%)	16 (6.5%)	24 (10.7%)	0.10
Hypertension, *n* (%)	220 (47.1%)	95 (38.9%)	125 (56.0%)	0.005
Chronic renal disease, *n* (%)	53 (11.3%)	6 (2.4%)	47 (21.0%)	< 0.001
COPD, *n* (%)	50 (10.7%)	31 (12.7%)	19 (8.5%)	0.14
Chronic liver disease, *n* (%)	31 (6.6%)	14 (5.7%)	17 (7.6%)	0.89
Active cancer, *n* (%)	69 (14.7%)	37 (15.1%)	32 (14.3%)	0.99
Organ dysfunction				
Septic shock (Sepsis-3), *n* (%) ^†^	127 (27.1%)	57 (23.3%)	70 (31.3%)	0.04
SAPS II^†^	51 (38–61)	48 (36–59)	53 (41–65)	< 0.001
SOFA at inclusion	8 (5–10)	6 (4–9)	9 (6–11)	< 0.001
SOFA at ICU discharge	1 (0–5)	0 (0–4)	1 (0–5)	0.22
ICU stay and organ support				
Duration of ICU stay, days	13 (8–22)	14 (9–22)	12 (8–22)	0.26
Mechanical ventilation, *n* (%)^†^	426 (91.2%)	231 (94.6%)	195 (87.4%)	0.41
Duration of mechanical ventilation, days	7 (4–14)	7 (4–14)	10 (5–16)	0.27
Vasopressors use, *n* (%)^†^	403 (86.2%)	202 (82.7%)	201 (90.1%)	0.003
RRT during ICU stay, *n* (%)	109 (23.3%)	25 (10.2%)	84 (37.6%)	< 0.001
Primary outcome				
One-year mortality, *n* (%)	115 (24.6%)	39 (16.0%)	76 (34.1%)	< 0.001
Secondary outcomes				
Duration of hospitalization after ICU discharge, days	11 (4–24)	11 (4–27)	12 (2–22)	0.68
Rehospitalization at 3 months, *n* (%)^§^	113 (36.5%)	54 (32.1%)	59 (41.5%)	0.086
SF-36 PCS at 3 months^‖^	40 (24–54)	41 (23–58)	36 (22–51)	0.43
SF-36 MCS at 3 months^‖^	45 (33–67)	45 (32–67)	44 (31–65)	0.80
Mortality at 3 months, *n* (%)^‡^	78 (16.7%)	21 (8.6%)	57 (25.7%)	< 0.001
Rehospitalization at 6 months, *n* (%)^¶^	131 (47.0%)	61 (40.1%)	70 (55.1%)	0.012
SF-36 PCS at 6 months^$^	44 (29–66)	47 (34–69)	37 (25–62)	0.009
SF-36 MCS at 6 months^$^	50 (27–77)	50 (21–74)	62 (31–91)	0.52
Mortality at 6 months, *n* (%)	92 (19.7%)	30 (12.3%)	62 (27.9%)	< 0.001
Rehospitalization at 12 months,				
* n* (%)^ǀ^	160 (50.3%)	87 (48.6%)	73 (52.5%)	0.48
SF-36 PCS at 12 months•	50 (31–74)	60 (37–81)	47 (23–63)	0.34
SF-36 MCS at 12 months•	58 (39–76)	43 (16–90)	28 (4–50)	0.22

Continuous variables were expressed as median (IQR) and were compared with the Mann–Whitney *U* test. Categorical variables were expressed as numbers (%) and were compared with the Fisher exact test or the Chi square test as appropriate

*ICU* intensive care unit, *BMI* body mass index, *COPD* chronic obstructive pulmonary disease, *SAPS II* Simplified Acute Physiologic Score, *SOFA* Sequential Organ Failure Assessment, *RRT* renal replacement therapy, *SF-36* short form-36 questionnaire, *PCS* physical component score, *MCS* mental component score, *IQR* interquartile range

^†^At inclusion

^‡^After ICU discharge, in-hospital deaths proportion during the same hospitalization at 3 months was 50% in subtype A versus 47% in subtype B (*p* = 0.79)

^§^Values calculated for 310 patients

^¶^Values calculated for 279 patients

^ǀ^Values calculated for 318 patients

No significant difference in missing information for readmission was found between subtypes A and B at 3, 6 and 12 months (Chi square test)

^‖^values calculated for 172 patients

^$^Values calculated for 169 patients

^•^Values calculated for 119 patients

No significant difference in SF-36 response rate was found between subtypes A and B at 3, 6 and 12 months (Chi square test)

Primary outcome is presented at one year after ICU discharge. Secondary outcomes are presented at 3 months, 6 months and 12 months after ICU discharge. A higher SF-36 score indicated a better mental and physical function

### Identification of sepsis classes at ICU discharge

Latent class analysis was used to create a two-class model of patients at ICU discharge, which we believe provided an optimal statistical fit to the data. Bayesian information criteria was the lowest in the two-class model in all the imputed datasets. Bayesian information criteria is an indicator of stronger model fit. A representative LCA model fit summary for one to four classes is provided in Additional file 1: Table S5.

At ICU discharge, 244 patients (52%) were assigned to subtype A and 223 patients (48%) were assigned to subtype B. The mean (standard deviation) posterior class membership probability was 0.94 (0.12) for subtype A and subtype B.

Using consensus *k* means clustering, we selected k = 2 as the optimal fit clustering solution for our population (Additional file 1: Fig. S2). The two identified classes of sepsis-survivors at ICU discharge using LCA and consensus *k* means clustering presented similar clinical patterns (Additional file 1: Fig. S2).

### Clinical and biomarker profiles of sepsis-survivor subtypes

**Fig. 2:**
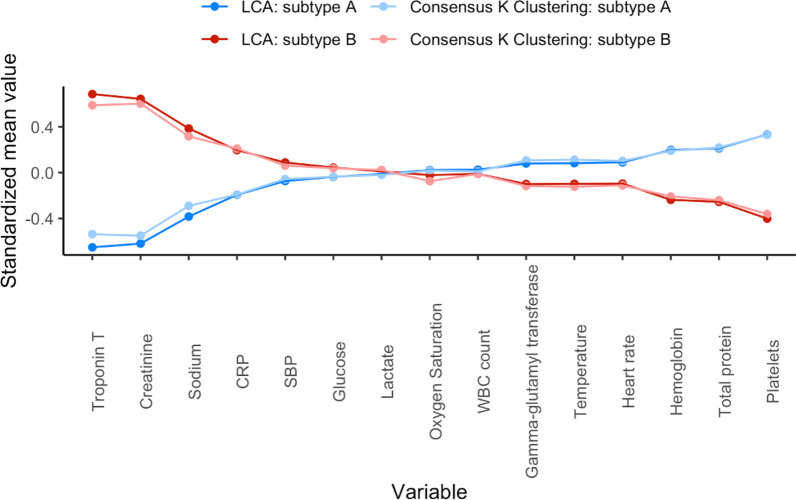
Comparison of class-defining variables using latent class analysis and consensus *k* means clustering. Description: continuous variables were plotted after natural log transformation. Every normalized variable was standardized such that all means are scaled to 0 and SDs to 1. Group means of standardized values are shown by subtype classes (A and B). A value of + 1 for the standardized variable (*y*-axis) indicates that the mean value for a given subtype was one SD higher than the mean value in the whole sepsis-survivors cohort (*N* = 467). Subtype classes sizes (*n*): Latent class analysis: subtype A *N* = 244, subtype B *N* = 223; consensus *k* means clustering: subtype A *N* = 255, subtype B *N* = 212 (concordance rate (accuracy) = 81%). The mean (± SD) of percent missingness of the 15 class-defining variables was 12% (± 6). No significant difference in missing information for class-defining variables was found between subtypes A and B at ICU discharge (Chi square test). Abbreviations: *SD* standard deviations, *BUN* blood urea nitrogen, *CRP* C-reactive protein, *SBP* systolic blood pressure, *WBC* white blood cell, *ICU* intensive care unit

The Patient characteristics and outcomes based on subtype classes are summarized in Table 1. Standardized mean difference plot of subtype-defining variables are shown in Fig. 2. Of the ICU discharge variables used in the LCA model, patients with subtype B (compared to subtype A) had: (i) higher circulating levels of serum creatinine levels, sodium, Troponin T levels and C-reactive protein; and (ii) lower circulating levels of platelets, hemoglobin and total protein compared to individuals in subtype A. Patients assigned to subtype B were mostly male, older, had more comorbidities, higher body mass index, were more severely ill on admission (i.e., higher Simplified Acute Physiology Score II (SAPS II) and SOFA scores) and had more bacteremia at inclusion. Sepsis-survivors subtype classes did not significantly differ by duration of ICU stay (Table 1).

**Fig. 3:**
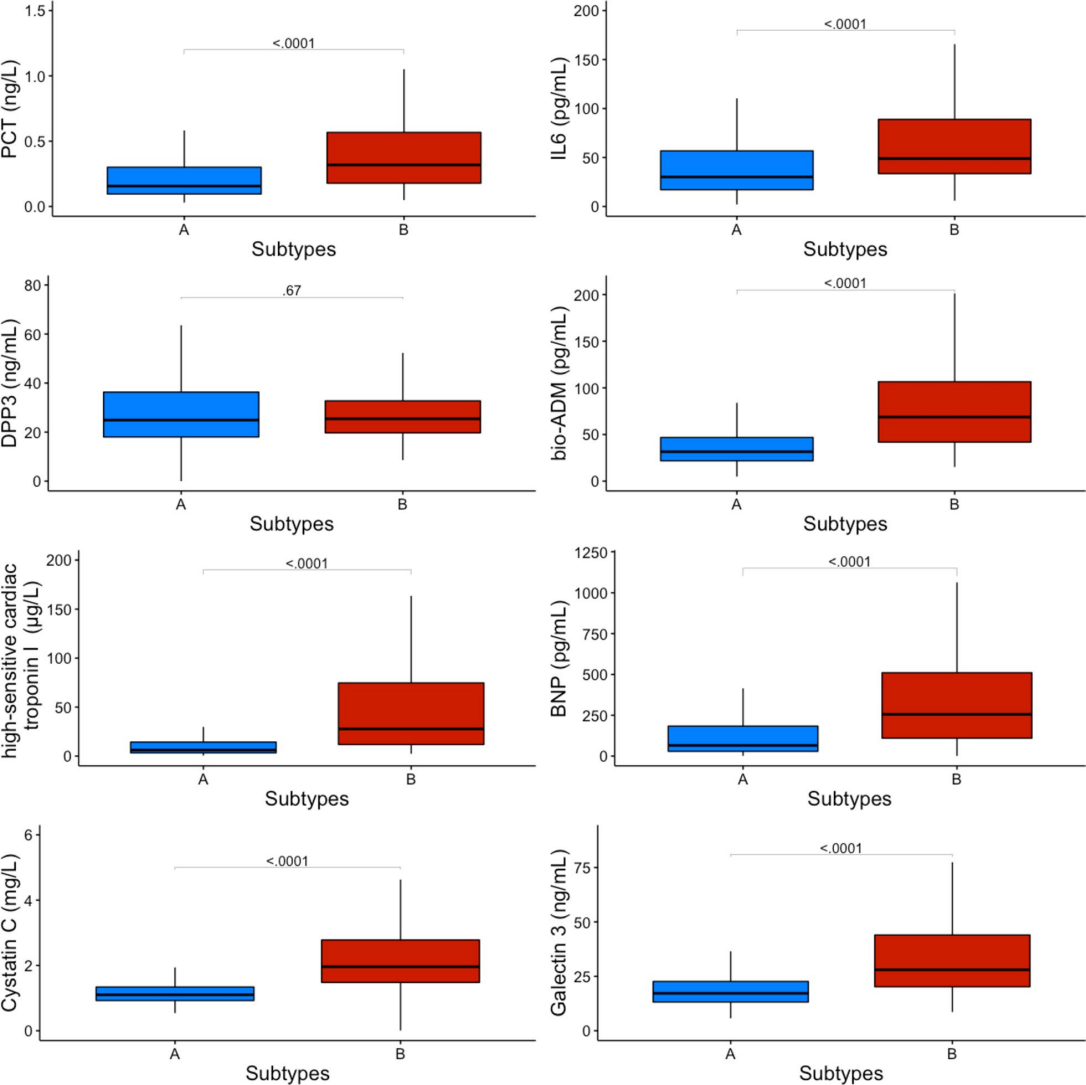
Comparison of host response biomarkers levels at ICU discharge between subtypes. Biomarkers data at ICU discharge were available for 350 patients (subtype A *N* = 191, subtype B *N* = 159). No significant difference in missing information for biomarkers at ICU discharge was found between subtypes A and B (Chi square test). Comparison for each biomarker was performed using the Mann–Whitney U test. Data are shown as median (IQR). Abbreviations: *ICU* intensive care unit, *PCT* procalcitonin, *IL6* interleukin-6, *DPP3* circulating dipeptidyl peptidase 3, *Bio-ADM* bio-adrenomedullin, *BNP* brain natriuretic peptide

To ascertain that sepsis-survivors subtypes represent subgroups of patients with underlying differential biology we measured and analyzed specific circulating mediators known to be associated with persistent inflammation and organ dysfunction and compared them between patients assigned to specific LCA-determined classes. Patients in subtype B demonstrated persistent elevation in levels of markers of inflammation (procalcitonin and interleukin-6), endothelial dysfunction (bio-adrenomedullin), myocardial injury and stress (high sensitivity cardiac troponin I, brain natriuretic peptide and galectin 3) and renal dysfunction (plasmatic cystatin C) compared to subtype A (Fig. 3). Circulating dipeptidyl peptidase 3 (DPP3) levels were similarly low in the two subtypes. Differences in circulating biomarker levels between sepsis-survivors subtypes persisted after subgroup analysis stratified by Charlson age–comorbidity index terciles, at discharge SOFA score terciles, on admission SAPS II terciles and sepsis severity on admission (Additional file 1: Figs. S3, S4, S5 and S6).

### Association between sepsis-survivors subtypes and outcomes

**Fig. 4:**
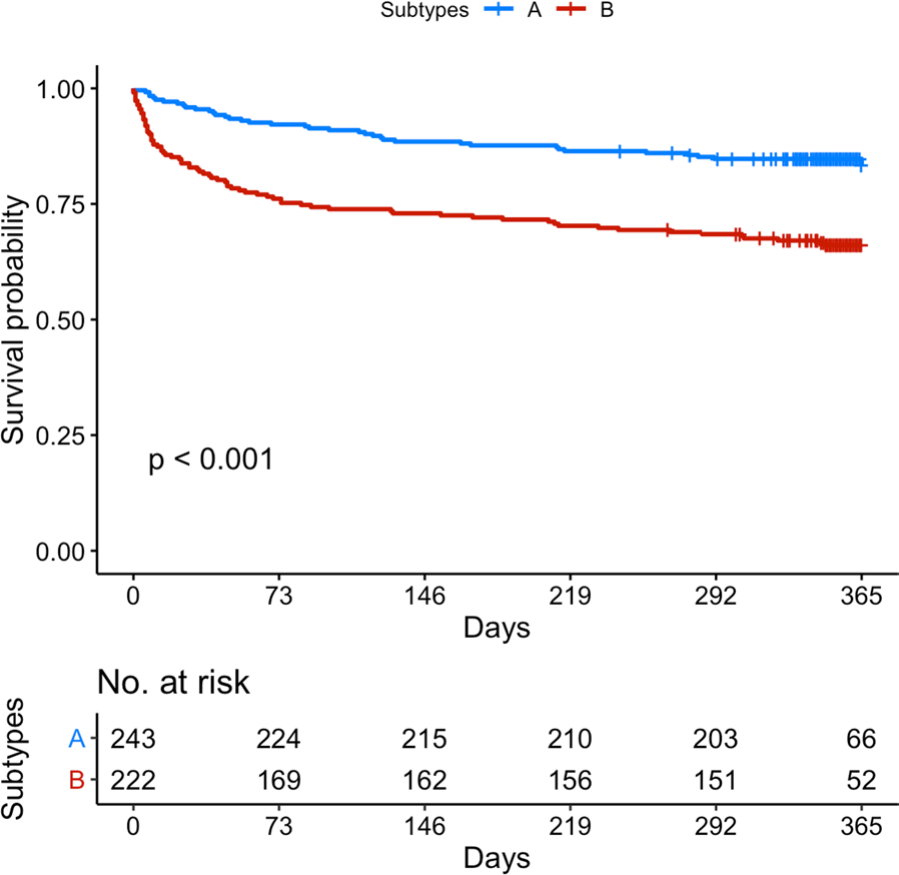
One-year post-ICU survival curves according to subtype membership. The log-rank test between the survival curves of the two subtypes at ICU discharge showed a *p* < 0.001

Differences between one-year survivors and non-survivors are summarized in Additional file 1: Table S6. Sepsis-survivors in subtype B had significantly higher one-year mortality compared to subtype A (respectively, 34% vs 16%, *p* < 0.001) (Table 1). The log-rank test between the one-year post-ICU survival curves of the two subtypes showed a *p* < 0.001 (Fig. 4). In a Cox proportional hazards model adjusted for Charlson age–comorbidity index, SAPS II at inclusion, SOFA score at ICU discharge and duration of ICU stay, membership in subtype B at ICU discharge was independently associated with one-year mortality (adjusted hazard ratio (HR) = 1.74 (95% CI 1.16–2.60); *p* = 0.006) (Table 2). In another Cox regression model adjusted for Charlson age–comorbidity index, SAPS II at inclusion, renal SOFA score at ICU discharge and duration of ICU stay, membership in subtype B at ICU discharge was also independently associated with one-year mortality (adjusted hazard ratio (HR) = 1.80 (95% CI 1.19–2.77); *p* = 0.005) (Additional file 1: Table 7). The same results were found when adjusting for age, chronic kidney disease, diabetes mellitus, SAPS II at inclusion, SOFA score at ICU discharge and duration of ICU stay (subtype B adjusted hazard ratio (HR) = 1.62 (95% CI 1.03–2.54); *p* = 0.03) (Additional file 1: Table 8). Sepsis-survivors in subtype B had significantly higher hospital readmissions at 6 months compared to subtype A (respectively, 55.1% vs 40.1%, *p* = 0.009). There was a significant difference between the two subtypes regarding SF-36 PCS at 6 months (i.e., lower physical quality of life in subtype B patients) but not at 3 and 12 months (Table 1).

**Table 2:** Cox proportional hazards models to adjust for confounding (Charlson age–comorbidity index, duration of ICU stay, SAPS II on admission, SOFA score at ICU discharge) for one-year mortality

	Adjusted HRs	CI 95%	*p* value
*Model with subtypes at ICU discharge*			
Harrell’s C-index = 0.73 (95% CI 0.69–0.77) Optimism < 0.01			
Subtype (A as reference)	1.74	(1.16–2.60)	0.006
Charlson age–comorbidity index	1.23	(1.14–1.33)	< 0.001
Duration of ICU stay (days)	1.00	(0.99–1.01)	0.31
SAPS II on admission (per 10-points increase)	1.07	(0.96–1.19)	0.18
SOFA score at ICU discharge	1.08	(1.02–1.13)	0.004
*Model without subtypes at ICU discharge*			
Harrell’s C-index = 0.71 (95% CI 0.68–0.76) Optimism < 0.01			
Charlson age–comorbidity index	1.25	(1.16–1.35)	< 0.001
Duration of ICU stay (days)	1.00	(0.99–1.01)	0.35
SAPS II on admission (per 10-points increase)	1.08	(0.97–1.20)	0.11
SOFA score at ICU discharge	1.08	(1.02–1.14)	0.002

After adjustment for Charlson age–comorbidity Index, duration of ICU stay, SAPS II on admission and SOFA score at ICU discharge, membership in subtype B at ICU discharge was independently associated with one-year mortality. The model calibration was good according to the Grønnesby–Borgan test (*p* = 0.66)

A significant improvement in Cox regression model discrimination was found when adding subtype membership at ICU discharge on top of Charlson age–comorbidity Index, duration of ICU stay, SAPS II on admission and SOFA score at ICU discharge with an increase in Harrell’s C-index by 2% (*p* = 0.006)

*HR* hazard ratio, *CI 95%* 95% confidence interval, *ICU* intensive care unit, *SAPS II* Simplified Acute Physiologic Score, *SOFA* Sequential Organ Failure Assessment

### Subtypes discrimination with reduced number of biomarkers

The initial multivariable regression model to discriminate the two classes was built with inflammatory and organ dysfunction biomarkers associated with at ICU discharge classes in univariate analysis. Thereafter, backward selection was applied to identify the main circulating markers associated with sepsis-survivors classes at ICU discharge. A reduced five-biomarker classification model including procalcitonin, plasmatic cystatin C, galectin 3, brain natriuretic peptide and bio-adrenomedullin measured at ICU discharge emerged (Additional file 1: Table 9).

## Discussion

Two sepsis-survivors classes were derived from 15 clinical and biological data available at the time of ICU discharge using an unsupervised analysis. When adjusted for standard risk factors (e.g., age, comorbidities, duration of ICU stay, severity of illness and renal function), subtype B was independently associated with increased one-year mortality after ICU discharge. This work was conducted in line with a recent 2018 colloquium on sepsis survivorship sponsored by the International Sepsis Forum suggesting a research road map to include phenotyping as an enrichment strategy to further understand heterogeneity among sepsis-survivors [4].

Several studies have used an unsupervised approach (i.e., phenotyping) including physiologic variables measured on admission to identify clinical subtypes of early sepsis with different outcomes [8, 9]. Nonetheless, only few studies used an unsupervised approach in sepsis-survivors.

In the study of Yende et al., two phenotypes of sepsis-survivors’ trajectories were identified in 483 patients using inflammation and immunosuppression biomarkers measured at five time points during and after hospitalization for sepsis for one year [11]. The hyperinflammation and immunosuppression phenotype was independently associated with higher one-year mortality when compared with the normal phenotype.

In Puthucheary et al. study, 291 adult sepsis-survivors were followed for 24 months. Physical function was the primary outcome and was mainly assessed using the PCS of the SF-36 [32]. Groups of longitudinal trajectories of PCS of the SF-36 were clustered using factor analysis. Two different physical recovery trajectories were identified. Older patients with more comorbidities and lower educational levels were more likely to have a poor physical recovery. A summary of these two studies is provided in Additional file 1: Table 10.

In the current study, sepsis-survivors assigned to subtype B had worse kidney function, were more anemic, had more coagulopathy and increased inflammation at ICU discharge when compared to subtype A. Furthermore, subtype B patients showed elevated markers of cardiovascular injury, though hemodynamically stable with low prognostic markers, including lactate and DPP3. Most of the circulating marker levels differences persisted between subtypes after subgroup analysis stratified by traditional patient groupings using Charlson age–comorbidity index terciles, at ICU discharge SOFA score terciles and sepsis syndrome severity on admission (i.e., SAPS II). When added to standard risk factors, subtype membership significantly improved post-ICU risk stratification and was independently associated with one-year mortality. Mortality at three and six months after ICU discharge was also significantly higher in sepsis-survivors assigned to subtype B. Hospital readmission rate was significantly higher in sepsis-survivors assigned to subtype B at six months. There was a significant difference between the two subtypes regarding SF-36 PCS at 6 months (i.e., lower physical quality of life in subtype B patients) but not at the other timepoints. A potential explanation of the mortality, readmissions and SF-36 PCS results differences at different timepoints after ICU discharge between subtypes A and B is that long-term mortality may be acting as a competing event for rehospitalization and physical disability as of 6 months after ICU discharge.

There are scarce data to elucidate mechanisms of long-term consequences of sepsis and how to optimize health post-sepsis. Our work suggests that persistent inflammation and worsening organ dysfunction (heart and kidney) in stabilized sepsis-survivors at ICU discharge may be associated with increased mortality and worsening underlying pathology. While persistent inflammation has been linked to accelerated atherosclerosis, plaque rupture and cardiovascular deaths; prolonged immunosuppression is probably related to post-sepsis syndrome associated infections [3, 34, 35].

Accordingly, identification of physiologic subtypes at ICU discharge may allow a better understanding of sepsis-survivors trajectory in the ICU (e.g., sustained organ dysfunction, hyperinflammation in stabilized patients) and improve the identification of patients at higher risk of poor long-term outcomes (e.g., physical disability and skeletal muscle dysfunction, readmissions, cardiovascular events, infection, death). This enrichment approach may increase the probability of identifying a treatment benefit in a given subtype. [36]. Moreover, modifiable risk factors at ICU discharge (e.g., anemia, hyperglycemia) as well as biomarker-guided interventions and follow-up using immunomodulation or cardiorenal protective treatments (e.g., renin–angiotensin–aldosterone system inhibitors) should be assessed in future sepsis-survivors trials with specific physiologic subtypes which are not necessarily associated with initial severity of illness. Cardiorenal and immune disorders could be positively modulated by renin–angiotensin–aldosterone system inhibition in stabilized sepsis-survivors at ICU discharge with potentially a reduction in the progression of organ dysfunction and an improvement in long-term outcomes [37–39].

While the major strength of our study is the availability of detailed data collected at ICU discharge and one-year outcomes linked to well annotated biological biospecimens collected at ICU discharge, our study has potential limitations. First, the study is from 2011 to 2013, and changes in the management of sepsis-survivors may have occurred in the interim. Second, the study was not externally validated as the performance of the different models wasn’t assessed in other external datasets. Nonetheless, large datasets with clinical and biomarker data at ICU discharge with a one-year follow-up are rare and no other studies were available for validation. Third, this study only included patients with sepsis within 24 h after inclusion. The original FROG-ICU study was not designed for a daily monitoring of sepsis criteria during ICU stay. Therefore, it was not possible to include patients who developed sepsis during their ICU stay because of unavailable data at the time of sepsis onset. Finally, the observational design of this study does not allow us to draw any firm conclusions regarding a causal relationship between subtypes membership and long-term outcome.

## Conclusion

In this reanalysis of the multicenter prospective FROG-ICU study, two distinct, and almost equally prevalent, physiologic subtypes were identified within sepsis-survivors using readily available clinical and biological data at ICU discharge. Mortality was higher in subtype B patients as of three months after ICU discharge. When adjusted for standard risk factors (e.g., age, comorbidities, severity of illness, renal function and duration of ICU stay), subtype B membership was independently associated with one-year mortality. Future sepsis-survivors adaptive trials using enrichment strategies (e.g., evaluation of subtype and biomarker-based treatment) should be designed. This will allow a more effective identification of therapeutic and prevention strategies to improve long-term outcome in sepsis-survivors.

We suggest the following roadmap in this exciting and growing post-critical care subtyping research: (i) To encourage subtyping centered on the biological/molecular drivers of the post-sepsis syndrome to identify and largely validate distinct mechanistic signatures in sepsis-survivors (i.e., endotypes). (ii) To develop an integrative subtyping approach using unsupervised machine learning and biomarker data to inform effective new therapies in future clinical trials. (iii) To set up trials with an innovative design (i.e., subtype-based trials) for a more effective drug assessment in sepsis-survivors.

## Supplementary Information

**Additional file 1. Methods**: Detailed description of statistical analysis. **Fig. S1**: Heatmap of correlation between selected variables for phenotyping. **Fig. S2**: Consensus k clustering results. **Fig. S3**: Comparison of host response biomarkers levels at ICU discharge between subtypes across different subgroups of Charlson age–comorbidity index terciles. **Fig. S4**: Comparison of host response biomarkers levels at ICU discharge between subtypes across different subgroups of at ICU discharge SOFA terciles. **Fig. S5**: Comparison of host response biomarkers levels at ICU discharge between subtypes across different subgroups of on admission SAPS II terciles. **Fig. S6**: Comparison of host response biomarkers levels at ICU discharge between subtypes across different subgroups of sepsis severity at inclusion. **Table S1**: Selected variables included in the LCA model. **Table S2**: Cardiovascular, inflammatory and renal biomarkers measured at ICU discharge. **Table S3**: Clinical and biological variables at ICU discharge based on subtypes. **Table S4**: Site of infection and microbiological differences between subtypes. **Table S5**: Comparison of LCA models at discharge with different numbers of classes in a representative imputed dataset. **Table S6**: Patients Characteristics’ according to one-year mortality after ICU discharge. **Table S7**: Cox proportional hazards models to adjust for confounding (age, chronic kidney disease, diabetes mellitus, duration of ICU stay, SAPS II on admission, SOFA score at ICU discharge) for one-year mortality. **Table S8**: Cox proportional hazards models to adjust for confounding (age, chronic kidney disease, diabetes mellitus, duration of ICU stay, SAPS II on admission, SOFA score at ICU discharge) for one-year mortality. **Table S9**: Initial and reduced biomarker regression models to discriminate the two subtypes at ICU discharge. **Table S10**: Characteristics of the main clinical studies using an unsupervised approach (i.e., phenotyping) to identify different classes in sepsis-survivors after ICU discharge.

## Data Availability

Requests for access to study data should be directed to the corresponding author for consideration and can be provided pending appropriate institutional review board approvals.

## Abbreviations

CI: Confidence interval
FROG-ICU: French and European Outcome Registry in Intensive Care Units
HR: Hazard ratio
ICU: Intensive care unit
IQR: Interquartile range
LCA: Latent class analysis
MCS: Mental component score
PCS: Physical component score
SAPS II: Simplified Acute Physiology Score II
SF-36: Short form-36 questionnaire
SOFA: Sequential Organ Failure Assessment
