# Supplementary material for: Identifying clinical subtypes in sepsis-survivors with different one-year outcomes: a secondary latent class analysis of the FROG-ICU cohort
Source: Crit Care. 2022 Apr 21;26:114. doi: 10.1186/s13054-022-03972-8 (PMC9022336; doi:10.1186/s13054-022-03972-8)
Supplement: Supplementary file 1 — Additional file 1. Methods: Detailed description of statistical analysis. Fig. S1: Heatmap of correlation between selected variables for phenotyping. Fig. S2: Consensus k clustering results. Fig. S3: Comparison of host response biomarkers levels at ICU discharge between subtypes across different subgroups of Charlson age–comorbidity index terciles. Fig. S4: Comparison of host response biomarkers levels at ICU discharge between subtypes across different subgroups of at ICU discharge SOFA terciles. Fig. S5: Comparison of host response biomarkers levels at ICU discharge between subtypes across different subgroups of on admission SAPS II terciles. Fig. S6: Comparison of host response biomarkers levels at ICU discharge between subtypes across different subgroups of sepsis severity at inclusion. Table S1: Selected variables included in the LCA model. Table S2: Cardiovascular, inflammatory and renal biomarkers measured at ICU discharge. Table S3: Clinical and biological variables at ICU discharge based on subtypes. Table S4: Site of infection and microbiological differences between subtypes. Table S5: Comparison of LCA models at discharge with different numbers of classes in a representative imputed dataset. Table S6: Patients Characteristics’ according to one-year mortality after ICU discharge. Table S7: Cox proportional hazards models to adjust for confounding (age, chronic kidney disease, diabetes mellitus, duration of ICU stay, SAPS II on admission, SOFA score at ICU discharge) for one-year mortality. Table S8: Cox proportional hazards models to adjust for confounding (age, chronic kidney disease, diabetes mellitus, duration of ICU stay, SAPS II on admission, SOFA score at ICU discharge) for one-year mortality. Table S9: Initial and reduced biomarker regression models to discriminate the two subtypes at ICU discharge. Table S10: Characteristics of the main clinical studies using an unsupervised approach (i.e., phenotyping) to identify different classes in sepsis-surviv [file 13054_2022_3972_MOESM1_ESM.docx]

**Additional file 1**

**Identifying clinical subtypes in sepsis-survivors with different one-year outcomes: A secondary latent class analysis of the FROG-ICU cohort**

Sabri Soussi, Divya Sharma, Peter Jüni, Gerald Lebovic, Laurent Brochard, John C. Marshall, Patrick R. Lawler, Margaret Herridge^,^ Niall Ferguson, Lorenzo Del Sorbo, Elodie Feliot, Alexandre Mebazaa, Erica Acton, Jason N Kennedy, Wei Xu, Etienne Gayat, Claudia C. dos Santos On behalf of the FROG-ICU and CCCTBG trans-trial group study for InFACT – the International Forum for Acute Care Trialists

|  | **Pages** |
| --- | --- |
| **e-Methods.** Detailed description of statistical analysis | 3 |
| **e-Figures** |  |
| **e-Figure1.** Heatmap of correlation between selected variables for phenotyping. | 5 |
| **e-Figure2.** Consensus *k* clustering results. | 6 |
| **e-Figure3.** Comparison of host response biomarkers levels at ICU discharge between subtypes across different subgroups of Charlson age-comorbidity index terciles | 8 |
| **e-Figure4.** Comparison of host response biomarkers levels at ICU discharge between subtypes across different subgroups of at ICU discharge SOFA terciles. | 9 |
| **e-Figure5.** Comparison of host response biomarkers levels at ICU discharge between subtypes across different subgroups of on admission SAPS II terciles. | 10 |
| **e-Figure6.** Comparison of host response biomarkers levels at ICU discharge between subtypes across different subgroups of sepsis severity at inclusion. | 11 |
| **e-Tables** |  |
| **e-Table1.** Selected variables included in the LCA model. | 12 |
| **e-Table2.** Cardiovascular, inflammatory and renal biomarkers measured at ICU discharge. | 13 |
| **e-Table3.** Clinical and biological variables at ICU discharge based on subtypes. | 14 |
| **e-Table4.** Site of infection and microbiological differences between subtypes. | 16 |
| **e-Table5.** Comparison of LCA models at discharge with different numbers of classes in a representative imputed dataset. | 17 |
| **e-Table6.** Patients Characteristics’ according to one-year mortality after ICU discharge. | 18 |
| **e-Table7.** Cox proportional hazards models to adjust for confounding (age, chronic kidney disease, diabetes mellitus, duration of ICU stay, SAPS II on admission, SOFA score at ICU discharge) for one-year mortality. | 19 |
| **e-Table8.** Cox proportional hazards models to adjust for confounding (age, chronic kidney disease, diabetes mellitus, duration of ICU stay, SAPS II on admission, SOFA score at ICU discharge) for one-year mortality. | 20 |
| **e-Table9.** Initial and reduced biomarker regression models to discriminate the two subtypes at ICU discharge. | 21 |
| **e-Table10.** Characteristics of the main clinical studies using an unsupervised approach (i.e., phenotyping) to identify different classes in sepsis-survivors after ICU discharge. | 22 |

**e-Methods. Detailed description of statistical analysis**

**Consensus k means clustering**

For internal validation of the latent classes identified with LCA, consensus k means clustering was used [1]. The optimal number of clusters was decided by considering clear separation of the consensus matrix heatmaps (i.e., highest class stability), characteristics of the consensus cumulative distribution function plots (i.e., the highest k model with cluster consensus values above 0.8 for all classes) and class size [2]. Final class assignment was determined by taking the majority votes in the 20 imputed datasets for each patient [3].

**Regression models**

Variables associated with at ICU discharge classes in univariate analysis were entered in a multivariate logistic regression model to identify a parsimonious set of biomarkers measured at discharge that could discriminate classes membership. Backward model selection algorithm was applied with stopping rules based on achieving the lowest Akaike Information Criterion (AIC) [4]. Variables with collinearity were excluded from the regression models (variance inflation factor >5 indicating the presence of multicollinearity) [5]. A rule of a minimum of 5-10 events for each predictor variable was considered [6]. Inference was combined from the sets of imputed samples using Rubin’s rules [7].

Cox proportional hazards and logistic regression models’ discriminations were respectively assessed using the Harrell’s C-statistic and receiver operating curve (ROC) analyses. To assess the incremental value (improvement in discrimination) of adding at discharge subtypes to a clinical Cox proportional hazards regression model with usual predictors of one-year outcome, the difference in the Harrell C-statistic between the aforesaid prediction models was assessed [8]. ROC curves derived from logistic regression models were compared using the Delong test [9]. For internal validation, a bootstrap resampling (2000 samples) was used to quantify any optimism within the different models (averaged difference between the apparent C-index or area under the curve (AUC) of the model developed on each bootstrap sample and its C-index or AUC on the original sample as appropriate) [10]. Calibration of the Cox proportional hazards and the selected biomarkers logistic regression models was respectively assessed using the Grønnesby-Borgan test and the Hosmer-Lemeshow test (i.e., goodness of fit) [11,12].

**References**

1. Wilkerson MD, Hayes DN. ConsensusClusterPlus: a class discovery tool with confidence assessments and item tracking. *Bioinformatics* 2010; 26:1572-3.

2. Seymour CW, Kennedy JN, Wang S, Chang C-CH, Elliott CF, Xu Z, et al. Derivation, Validation, and Potential Treatment Implications of Novel Clinical Phenotypes for Sepsis. JAMA. 2019;321:2003–17.

3. Basagaña X, Barrera-Gómez J, Benet M, Antó JM, Garcia-Aymerich J. A framework for multiple imputation in cluster analysis. Am J Epidemiol. 2013;177:718–25.

4. Sanchez-Pinto LN, Venable LR, Fahrenbach J, Churpek MM. Comparison of variable selection methods for clinical predictive modeling. Int J Med Inform. 2018;116:10–7.

5. Vatcheva KP, Lee M, McCormick JB, Rahbar MH. Multicollinearity in Regression Analyses Conducted in Epidemiologic Studies. *Epidemiology (Sunnyvale)* 2016; 6:227.

6. Vittinghoff E, McCulloch CE. Relaxing the rule of ten events per variable in logistic and Cox regression. Am J Epidemiol. 2007;165:710–8.

7. Marshall A, Altman DG, Holder RL, Royston P. Combining estimates of interest in prognostic modelling studies after multiple imputation: current practice and guidelines. BMC Med Res Methodol. 2009;9:57.

8. Kang L, Chen W, Petrick NA, Gallas BD. Comparing two correlated C indices with right-censored survival outcome: a one-shot nonparametric approach. Stat Med. 2015;34:685–703.

9. Seshan VE, Gönen M, Begg CB. Comparing ROC curves derived from regression models. Stat Med. 2013;32:1483–93.

10. Collins GS, Reitsma JB, Altman DG, Moons KGM, TRIPOD Group. Transparent reporting of a multivariable prediction model for individual prognosis or diagnosis (TRIPOD): the TRIPOD statement. The TRIPOD Group. Circulation. 2015;131:211–9.

11. Demler OV, Paynter NP, Cook NR. Tests of calibration and goodness-of-fit in the survival setting. Stat Med. 2015;34:1659–80.

12. Soussi S, Collins GS, Jüni P, Mebazaa A, Gayat E, Le Manach Y. Evaluation of Biomarkers in Critical Care and Perioperative Medicine: A Clinician’s Overview of Traditional Statistical Methods and Machine Learning Algorithms. Anesthesiology. 2021;134:15–25.

**e-Figure 1. Heatmap of correlation between selected variables for phenotyping (*N*=15).**


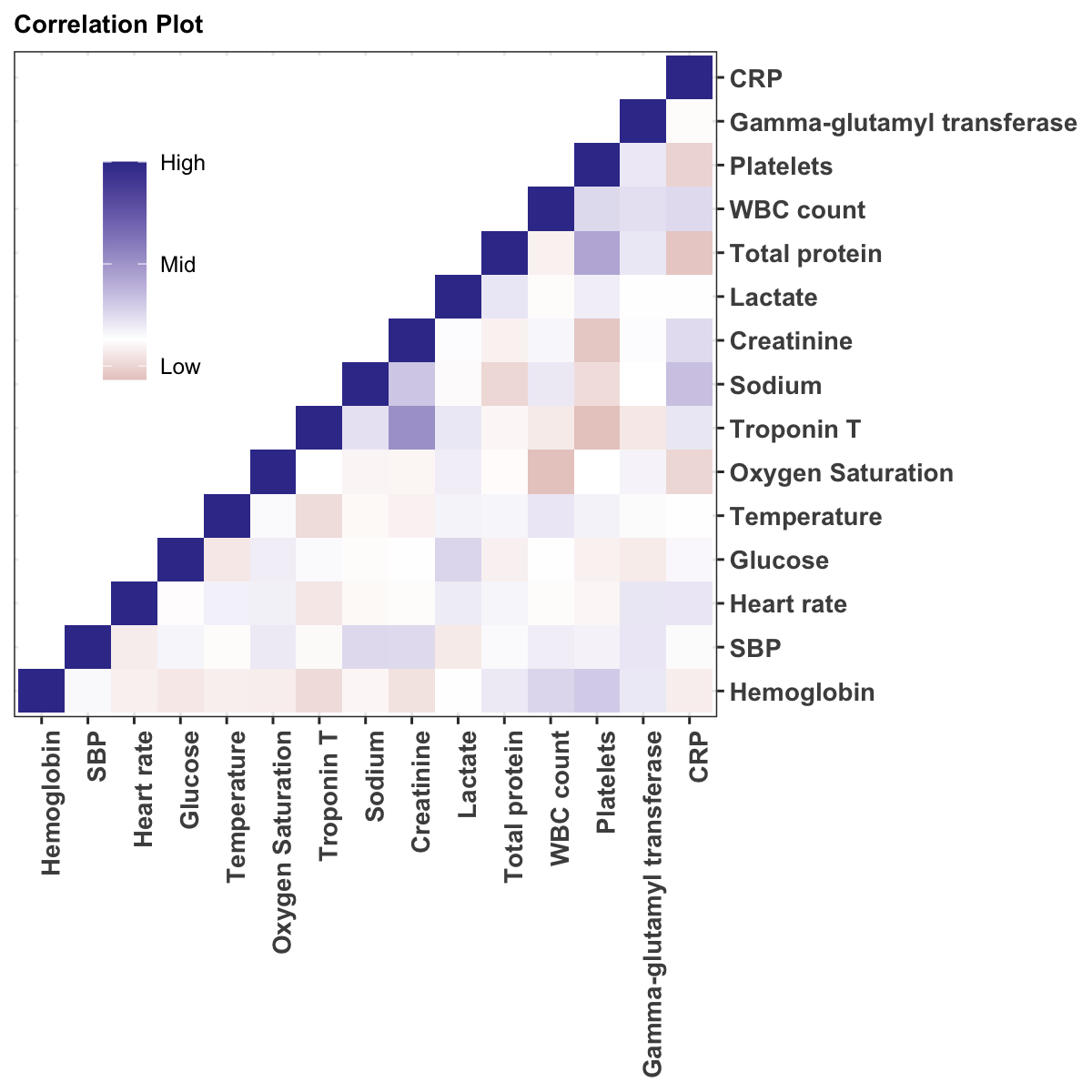


Heatmap shows greater color (blue or pink) when the Spearman’s rank correlation coefficient is greater in positive or negative direction.

Abbreviations: CRP, C-reactive protein; SBP, systolic blood pressure; WBC, white blood cell.

**e-Figure 2 Consensus *k* clustering results**.

**(A)**

**
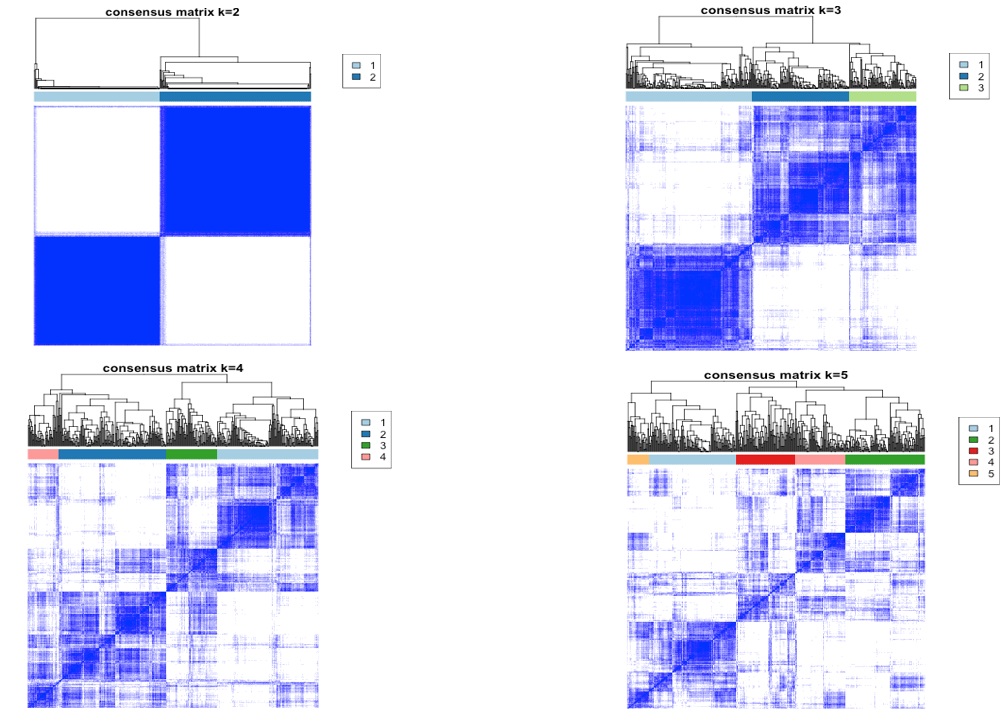
**

(A) Representative heatmaps illustrating consensus k-means clustering. Unsupervised consensus *k* clustering showed highest class stability in consensus matrix for *k*=2 suggesting that two may be the optimal choice for the number of classes. Subtype classes sizes (n): subtype A n= 255, subtype B n=212.

**(B)**


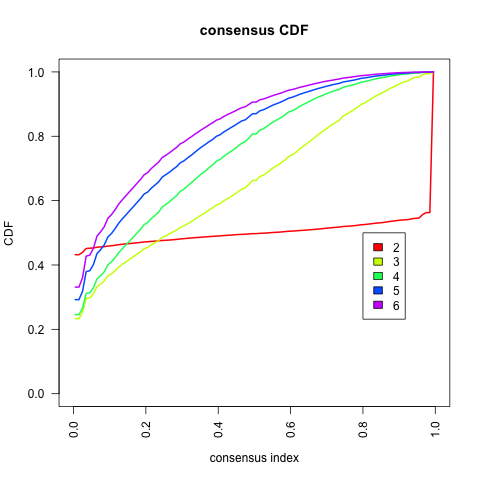


(B) Consensus cumulative distribution function (CDF) plot across two to six classes (k). More horizontal curves suggest optimal fit (k=2).

**(C)**


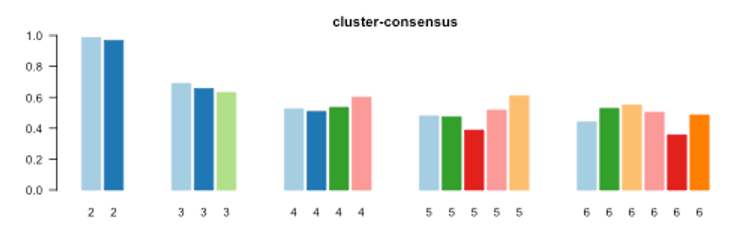


(C) Cluster consensus plot showing the mean of all pairwise consensus values between a cluster member, for two to six classes (k). Higher values for all bars suggest optimal fit (cluster consensus values above 0.8 for all classes).

**e-Figure 3 Comparison of host response biomarkers levels at ICU discharge between subtypes across different subgroups of Charlson age-comorbidity index terciles**

Terciles were set up based on Charlson age-comorbidity indexes and age. When Charlson age-comorbidity indexes were equal, we considered age to classify the patients into the different terciles.

Subgroups’ sizes (*N*): 1^st^ Tercile: Subtype A *N*= 84, Subtype B *N*= 32; 2^nd^ Tercile: Subtype A *N*= 69, Subtype B *N*= 55; 3^rd^ Tercile: Subtype A *N*= 38, Subtype B *N*= 72.

Comparison for each biomarker within subgroups was performed using the Mann-Whitney U test. Data are shown as median (IQR).

Abbreviations: ICU, Intensive care unit; PCT, procalcitonin; IL6, interleukin-6; DPP3, circulating dipeptidyl peptidase 3; Bio-ADM, bio-adrenomedullin; BNP, brain natriuretic peptide.


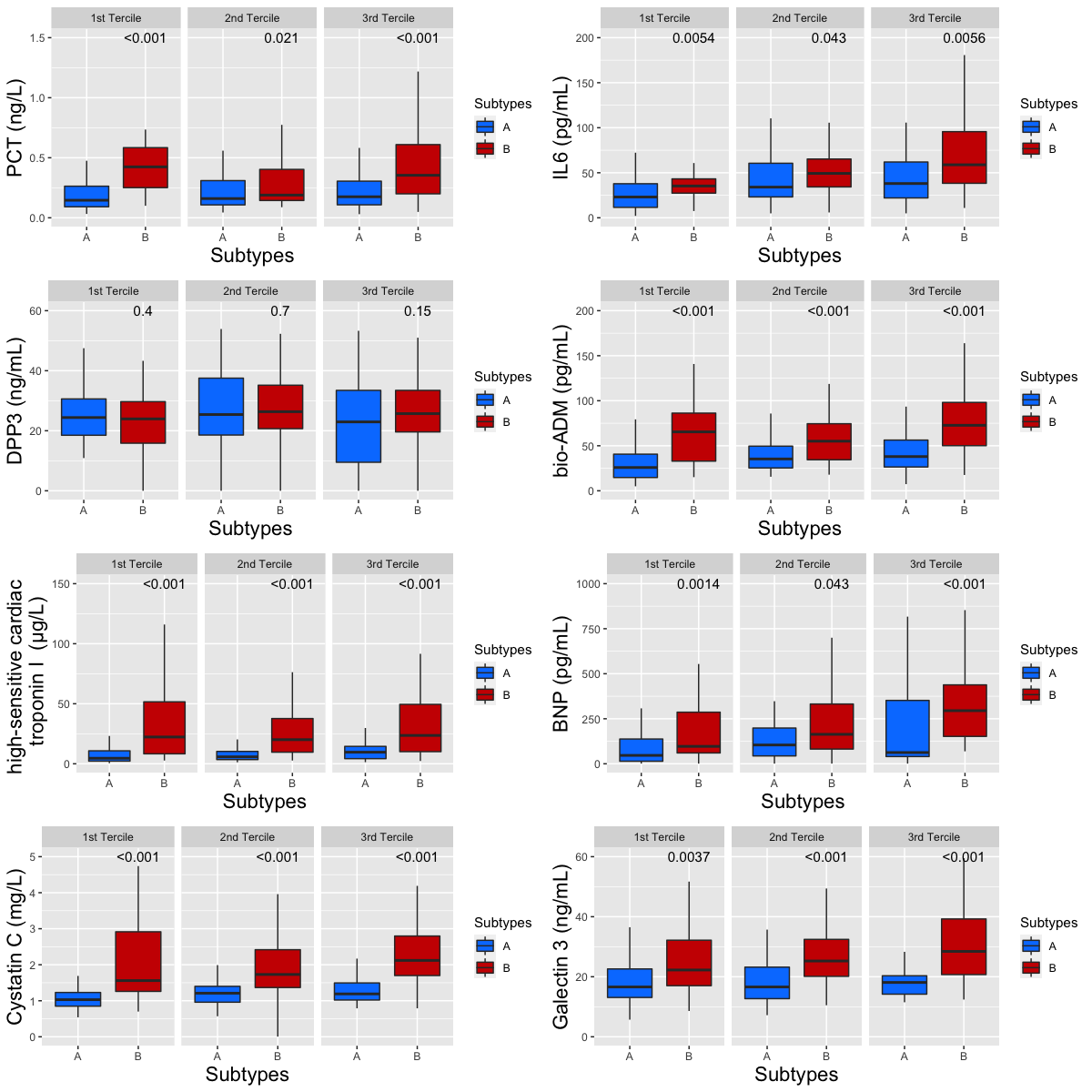


**e-Figure 4 Comparison of host response biomarkers levels at ICU discharge between subtypes across different subgroups of at ICU discharge SOFA terciles.**

Terciles were set up based on available SOFA scores and creatinine levels at ICU discharge. When SOFA scores at ICU discharge were equal, we considered creatinine levels at ICU discharge to classify the patients into the different terciles.

Subgroups’ sizes (*N*): 1^st^ Tercile: Subtype A *N*= 75, Subtype B *N*= 25; 2^nd^ Tercile: Subtype A *N*= 33, Subtype B *N*= 62; 3^rd^ Tercile: Subtype A *N*= 45, Subtype B *N*= 45.

Comparison for each biomarker within subgroups was performed using the Mann-Whitney U test. Data are shown as median (IQR).

Abbreviations: ICU, Intensive care unit; PCT, procalcitonin; IL6, interleukin-6; DPP3, circulating dipeptidyl peptidase 3; Bio-ADM, bio-adrenomedullin; BNP, brain natriuretic peptide.


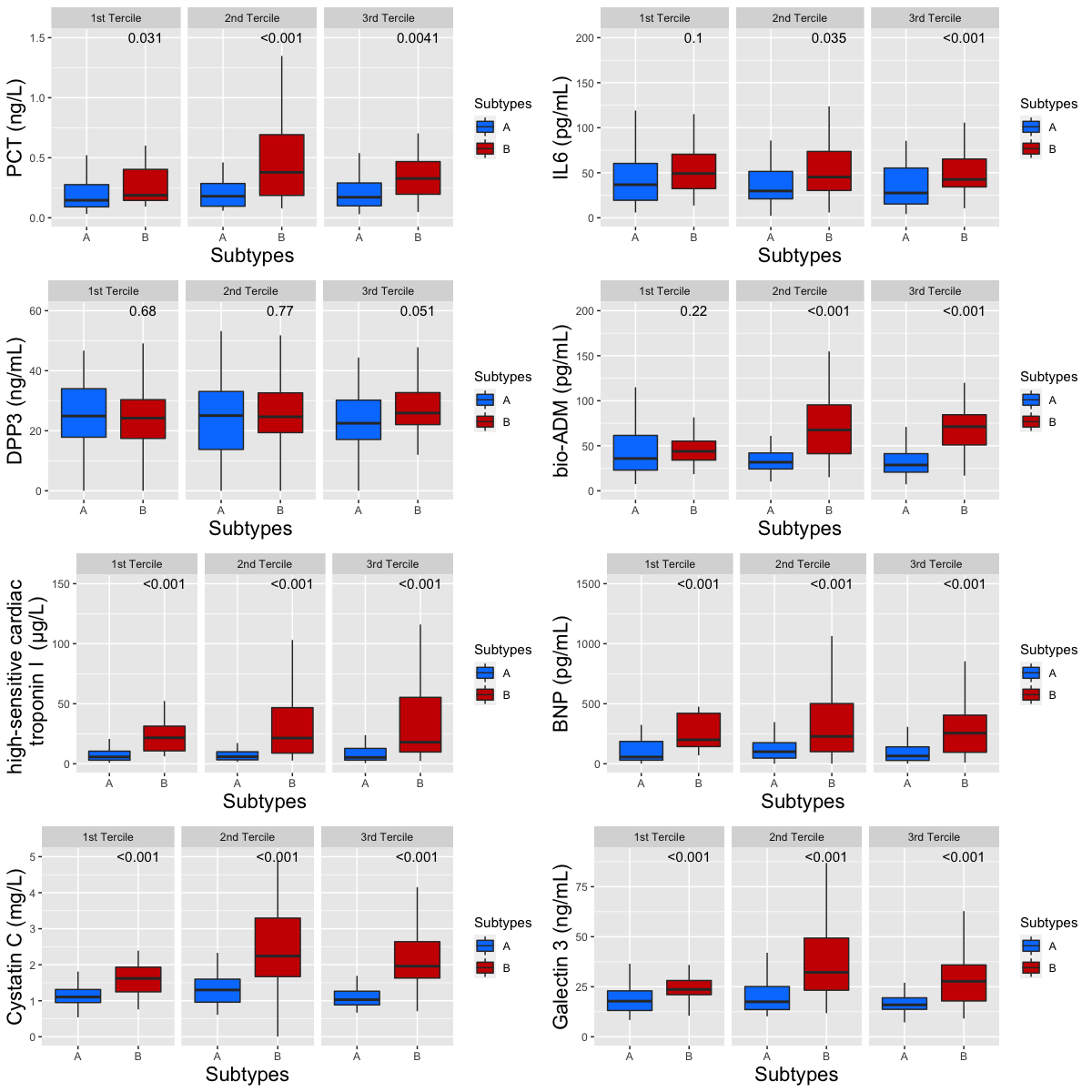


**e-Figure 5 Comparison of host response biomarkers levels at ICU discharge between subtypes across different subgroups of on admission SAPS II terciles.**

Terciles were set up based on SAPS II and age. When SAPS II were equal, we considered age to classify the patients into the different terciles.

Subgroups’ sizes (*N*): 1^st^ Tercile: Subtype A *N*= 82, Subtype B *N*= 40; 2^nd^ Tercile: Subtype A *N*= 67, Subtype B *N*= 58; 3^rd^ Tercile: Subtype A *N*= 42, Subtype B *N*= 61.

Comparison for each biomarker within subgroups was performed using the Mann-Whitney U test. Data are shown as median (IQR).

Abbreviations: ICU, Intensive care unit; SAPS II, Simplified Acute Physiologic Score; PCT, procalcitonin; IL6, interleukin-6; DPP3, circulating dipeptidyl peptidase 3; Bio-ADM, bio-adrenomedullin; BNP, brain natriuretic peptide.


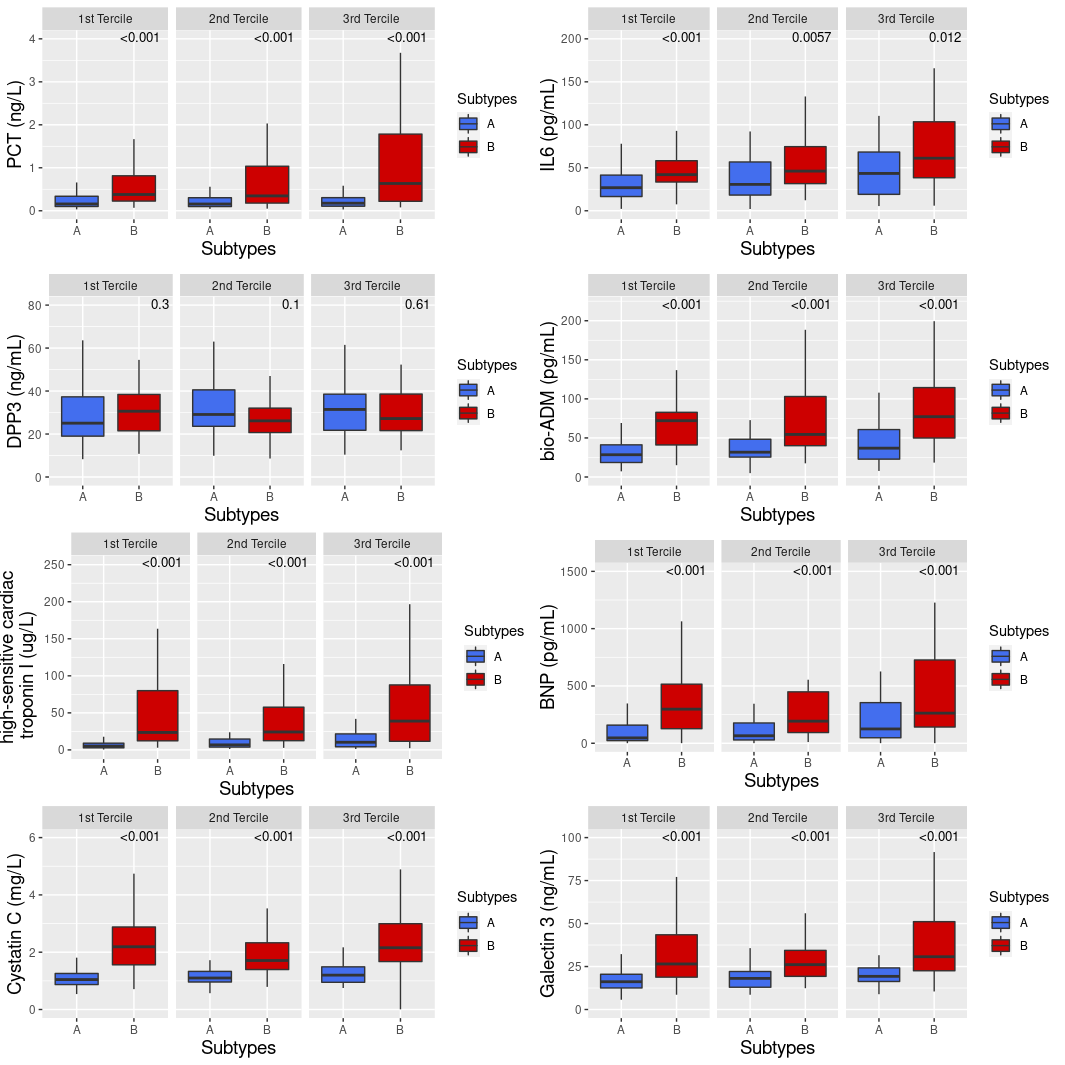


**e-Figure 6 Comparison of host response biomarkers levels at ICU discharge between subtypes across different subgroups of sepsis severity at inclusion (i.e., sepsis without vasopressors, sepsis with vasopressors and septic shock).**

Subgroups’ sizes (*N*): SWV: Subtype A *N*= 21, Subtype B *N*= 11; SV: Subtype A *N*= 136, Subtype B *N*= 105; SS: Subtype A *N*= 34, Subtype B *N*= 43.

Comparison for each biomarker within subgroups was performed using the Mann-Whitney U test. Data are shown as median (IQR).

Abbreviations: ICU, Intensive care unit; SWV, sepsis without vasopressors; SV, sepsis with vasopressors; SS, septic shock; PCT, procalcitonin; IL6, interleukin-6; DPP3, circulating dipeptidyl peptidase 3; Bio-ADM, bio-adrenomedullin; BNP, brain natriuretic peptide.


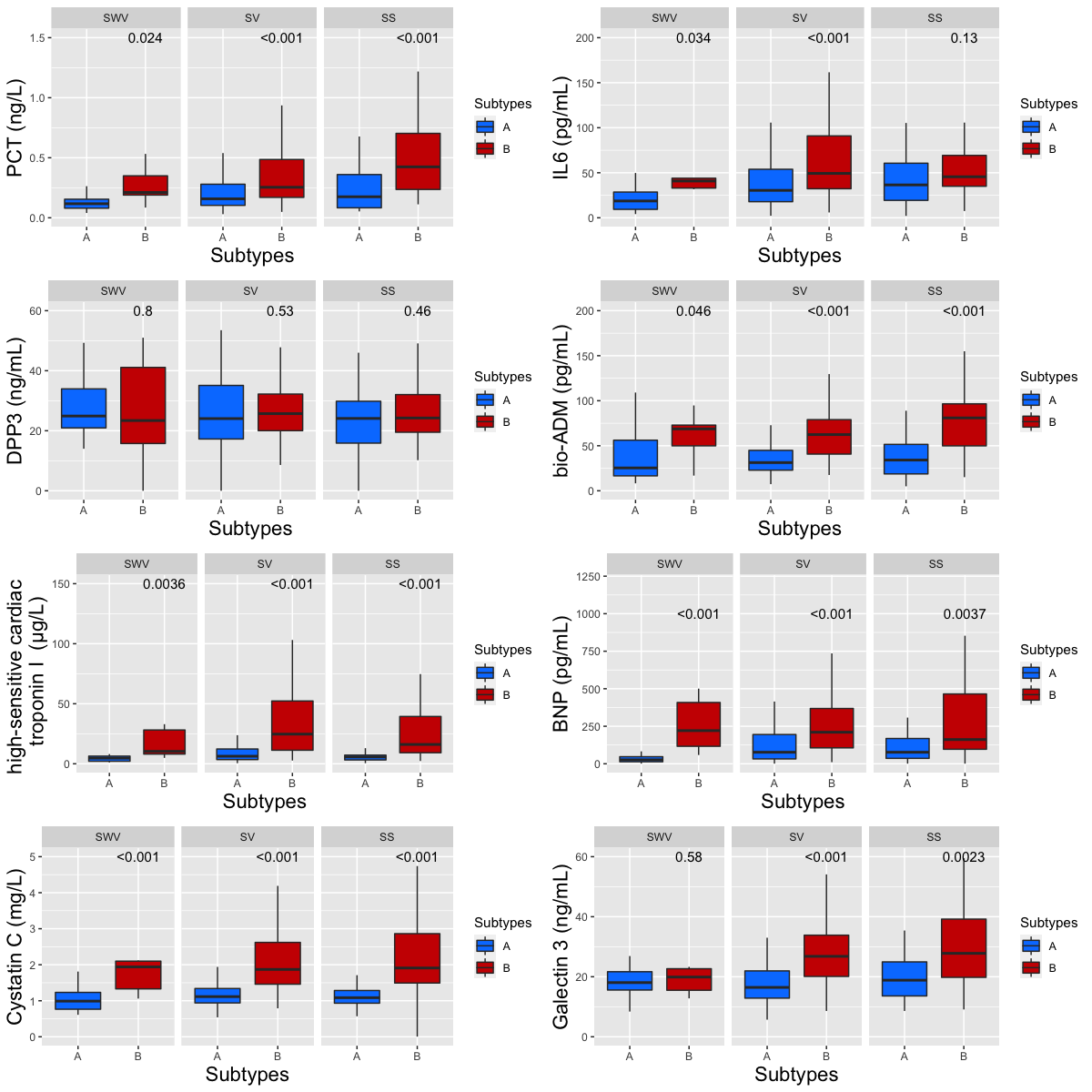


**e-Table1 Selected variables included in the LCA model.**

| **Variables at ICU discharge (*N*=15)** | **% of Missing values** | **Directionality of abnormal values** |
| --- | --- | --- |
| CRP | 24% | Maximum |
| Creatinine | 3% | Maximum |
| Gamma-glutamyl transferase | 15% | Minimum |
| Glucose | 20% | Maximum |
| Heart rate | 12% | Maximum |
| Hemoglobin | 11% | Minimum |
| Lactate | 14% | Maximum |
| Oxygen saturation | 11% | Minimum |
| Platelets | 10% | Minimum |
| Sodium | 2% | Maximum |
| SBP | 12% | Minimum |
| Temperature | 11% | Maximum |
| Troponin T | 24% | Maximum |
| Total protein | 5% | Maximum |
| WBC count | 10% | Maximum |

Variables are cited in alphabetical order. For each variable, the most abnormal value within the last 48 hours before ICU discharge was extracted.

Variables considered but excluded due to missingness (>25%): plasma bilirubin and albumin at ICU discharge.

Abbreviations: LCA, latent class analysis; ICU, Intensive care unit; CRP, C-reactive protein; SBP, systolic blood pressure; WBC, white blood cell.

**e-Table 2. Cardiovascular, inflammatory and renal biomarkers measured at ICU discharge**

| **Biomarkers** | **Description** |
| --- | --- |
| Cardio-vascular biomarkers | |
| High-sensitivity Troponin I | -Part of troponin complex, heart contraction.  Marker of cardiomyocyte injury. |
| Brain natriuretic peptide (BNP) | -Increase of natriuresis and decrease of vascular resistance.  Marker of cardiomyocyte stress. |
| Bio-adrenomedullin (bio-ADM) | -Vasodilatation, induction of angiogenesis, antimicrobial activity, protection against oxidative stress and hypoxic injury.  Marker of endothelial dysfunction. |
| Galectin 3 | -Involved in inflammation, fibrosis and neoplastic transformation.  Marker of heart failure. |
| Dipeptidyl peptidase 3 (DPP3) | -A metallopeptidase (mainly intracellular) involved in the metabolism of cardiovascular and inflammatory mediators that exert a direct negative inotropic action.  Prognostic marker in acute phase of shock. |
| Biomarkers of infection and/or inflammation | |
| Interleukin-6 | -Pleotropic cytokine with both pro- and anti-inflammatory activity, endogenous pyrogen involved in B cell maturation.  Marker of inflammation. |
| Procalcitonin (PCT) | -Precursor of calcitonin.  Marker of infection, mostly bacterial. |
| Renal biomarker | |
| Cystatin C | -Ubiquitously expressed inhibitor of lysosomal and cysteine proteinases (prevents the breakdown of proteins outside the cell), reabsorbed by proximal tubular cells.  Marker of decrease glomerular filtration rate. |

Inspired by Mebazaa A, Casadio MC, Azoulay E, et al. Post-ICU discharge and outcome: rationale and methods of the The French and euRopean Outcome reGistry in Intensive Care Units (FROG-ICU) observational study. BMC Anesthesiol 2015; 15:143.

**e-Table 3 Clinical and biological variables at ICU discharge based on subtypes.**

|  | **All patients**  **(*N*=467)** | **Subtype A**  **(*N*=244)** | **Subtype B**  **(*N*=223)** | ***P* value** |
| --- | --- | --- | --- | --- |
| **Inflammation** |  |  |  |  |
| Temperature, °C | 37.1 (36.7-37.5) | 37.1 (36.8-37.5) | 37.0 (36.6-37.4) | 0.018 |
| White blood cell count, ×10^9^/L | 10.0 (7.4-13.9) | 10.0 (7.3-14.0) | 9.9 (7.5-13.7) | 0.5 |
| C-reactive protein, mg/L | 60 (32-104) | 53 (28-90) | 75 (38-127) | 0.006 |
| **Cardiovascular or hemodynamic** |  |  |  |  |
| Heart rate, beats/min | 91 (79-103) | 93 (83-103) | 87 (75-103) | 0.02 |
| Systolic blood pressure, mm Hg | 124 (111-140) | 123 (111-136) | 126 (112-143) | 0.17 |
| Lactate, mmol/L | 1.3 (1.0-1.9) | 1.3 (0.9-1.9) | 1.4 (1.0-1.9) | 0.54 |
| Troponin T, ng/L | 33 (17-72) | 19 (11-29) | 71 (42-157) | <0.001 |
| **Pulmonary** |  |  |  |  |
| Respiratory rate, breaths/min | 22 (18-26) | 22 (18-26) | 22 (18-27) | 0.51 |
| Oxygen saturation, % | 97 (95-99) | 97 (95-100) | 97 (95-99) | 0.15 |
| Partial pressure of oxygen,  mm Hg | 77 (67-90) | 79 (67-90) | 76 (67-89) | 0.23 |
| **Renal** |  |  |  |  |
| Blood urea nitrogen, mmol/L | 7.3 (4.7-11.8) | 5.6 (4.0-7.4) | 11.0 (7.2-16.8) | <0.001 |
| Creatinine, μmol/L | 69 (52-110) | 54 (43-69) | 103 (73-197) | <0.001 |
| **Hepatic** |  |  |  |  |
| Alanine transaminase, U/L | 36 (21-72) | 36 (21-84) | 35 (20-57) | 0.33 |
| Aspartate transaminase, U/L | 38 (24-57) | 38 (25-55) | 39 (23-61) | 0.8 |
| Bilirubin, μmol/L | 11 (7-22) | 10 (6-18) | 13 (8-32) | 0.01 |
| Gamma-glutamyl transferase, U/L | 130 (57-229) | 139 (69-259) | 112 (47-205) | 0.005 |
| **Hematologic** |  |  |  |  |
| Hemoglobin, g/dL | 9.7 (8.7-10.7) | 10.0 (9.1-10.9) | 9.4 (8.4-10.5) | 0.002 |
| Platelets, ×10^9^/L | 301 (178-442) | 385 (229-534) | 236(150-335) | <0.001 |
| Prothrombin time, % | 74 (63-84) | 75 (65-87) | 71 (62-82) | 0.06 |
| **Neurologic** |  |  |  |  |
| Glasgow Coma Scale score | 15 (15-15) | 15 (15-15) | 15 (15-15) | 0.11 |
| **Metabolic** |  |  |  |  |
| Sodium, mmol/L | 139 (135-142) | 137 (135-140) | 141 (137-144) | <0.001 |
| Chloride, mmol/L | 98 (94-102) | 98 (93-102) | 99 (95-104) | 0.03 |
| Bicarbonate, mmol/L | 26 (23-29) | 26 (24-29) | 25 (22-28) | 0.006 |
| Glucose, mmol/L | 7.1 (5.9-8.3) | 7.0 (6.0-8.2) | 7.2 (5.9-8.6) | 0.26 |
| Total protein, g/L | 61 (54-67) | 63 (57-71) | 58 (51-64) | <0.001 |

Continuous variables were expressed as median (IQR) and were compared with the Mann-Whitney U test. Categorical variables were expressed as numbers (%) and were compared with the Fisher exact test or the Chi square test as appropriate.

All laboratory values are the maximum or minimum value within 48 hours before patients discharge from ICU.

Abbreviations: ICU, intensive care unit; IQR, interquartile range.

**e-Table 4 Site of infection and microbiological differences between subtypes**

|  | **All patients**  **(*N*=467)** | **Subtype A**  **(*N*=244)** | **Subtype B**  **(*N*=223)** | ***P* value** |
| --- | --- | --- | --- | --- |
| **Site of infection** |  |  |  | 0.37 |
| Pulmonary, n (%) | 191 (40.8%) | 106 (43.4%) | 85 (38.1%) |  |
| Abdominal, n (%) | 142 (30.4%) | 73 (29.9%) | 69 (30.9%) |  |
| Urinary, n (%) | 30 (6.4%) | 14 (5.7%) | 16 (7.1%) |  |
| Blood, n (%) | 23 (4.9%) | 11 (4.5%) | 12 (5.3%) |  |
| Wound and soft tissue, n (%) | 30 (6.4%) | 16 (6.6%) | 14 (6.3%) |  |
| Central nervous system, n (%) | 7 (1.5%) | 6 (2.5%) | 1 (0.4%) |  |
| Other, n (%) † | 26 (5.6%) | 12 (4.9%) | 14 (6.3%) |  |
| Unknown, n (%) | 18 (3.8%) | 8 (2.8%) | 10 (5.2%) |  |
| **Microorganisms ‡** |  |  |  |  |
| Gram positive, n (%) | 181 (50.0%) | 93 (49.7%) | 88 (50.3%) | 0.91 |
| Gram negative, n (%) | 232 (64.1%) | 122 (65.2%) | 110 (62.9%) | 0.63 |
| Candida, n (%) | 20 (5.5%) | 12 (3.3%) | 8 (2.2%) | 0.47 |
| Other organisms, n (%) | 10 (2.8%) | 7 (3.7%) | 3 (1.7%) | 0.23 |
| Bacteremia, n (%) | 105 (22.4%) | 48 (19.6%) | 57 (25.5%) | 0.002 |
| Supra-infection during ICU stay, n (%) | 244 (52.2%) | 130 (53.2 %) | 114 (51.1%) | 0.8 |

Categorical variables were expressed as numbers (%) and were compared with the Fisher exact test or the Chi square test as appropriate.

† Osteoarticular, pleural and mediastinal

‡ Data available for n=362 patients; Not mutually exclusive

Abbreviations: ICU, intensive care unit.

**e-Table 5 Comparison of LCA models at discharge with different numbers of classes in a representative imputed dataset**

|  |  | **Class size** | | | |
| --- | --- | --- | --- | --- | --- |
| **Class number** | **BIC** | **1** | **2** | **3** | **4** |
| **1** | 19961 | 467 | - | - | - |
| **2*** | 19898 | 244 | 223 | - | - |
| **3** | 19987 | 199 | 185 | 83 | - |
| **4** | 20079 | 200 | 127 | 93 | 47 |

Bayesian information criterion (BIC) is a metric for model selection. Lower values suggest model parsimony (i.e., simple models with high predictive power).

*Selected two-class LCA model.

BIC was the lowest in the two-class model in all the imputed datasets (20 imputed datasets).

The median (IQR) class sizes across the 20 imputed datasets were 235 (224-251) for class A and 232 (215-242) for class B (selected two-class models).

Abbreviations: LCA, Latent class analysis; BIC, Bayesian information criteria, IQR, Interquartile range.

**e-Table 6 Patients Characteristics’ according to one-year mortality after ICU discharge**

|  | **Survivors at one year**  **(*N*=352)** | **Non-survivors at one year**  **(*N*=115)** | ***P* value** |
| --- | --- | --- | --- |
| Age, years † | 48 (36-59) | 57 (44-67) | <0.001 |
| Male gender | 220 (62.5%) | 73 (63.4%) | 0.93 |
| BMI, Kg/m² † | 27 (23-31) | 27 (23-30) | <0.001 |
| **Comorbidities** † |  |  |  |
| Charlson age-comorbidity index | 3 (1-4) | 5 (3-6) | <0.001 |
| Diabetes mellitus, n (%) | 64 (18.1%) | 34 (29.5%) | 0.013 |
| Chronic heart failure, n (%) | 16 (4.5%) | 19 (16.5%) | <0.001 |
| Coronary artery disease, n (%) | 28 (7.9%) | 12 (10.4%) | 0.52 |
| Hypertension, n (%) | 152 (43.1%) | 68 (59.1%) | 0.004 |
| Dyslipidemia, n (%) | 68 (19.3%) | 27 (23.4%) | 0.40 |
| chronic renal disease, n (%) | 27 (7.6%) | 26 (22.6%) | <0.001 |
| COPD, n (%) | 37 (10.5%) | 13 (11.3%) | 0.94 |
| Chronic liver disease, n (%) | 17 (4.8%) | 14 (12.1%) | 0.011 |
| Active cancer, n (%) | 44 (12.5%) | 25 (21.7%) | 0.023 |
| **Organ dysfunction** † |  |  |  |
| SAPS II | 48 (36-59) | 57 (44-67) | <0.001 |
| SOFA | 7 (4-10) | 8 (6-11) | 0.017 |
| Septic shock (Sepsis-3), n (%) | 91 (25.8%) | 36 (31.3%) | 0.30 |
| **ICU stay and organ support** |  |  |  |
| Duration of ICU stay, days | 13 (8-22) | 14 (9-25) | 0.33 |
| Mechanical ventilation, n (%) † | 327 (92.8%) | 99 (86.0%) | 0.04 |
| Duration of mechanical ventilation, days | 7 (4-13) | 7 (5-14) | 0.43 |
| Tracheostomy, n (%) | 35 (9.9%) | 12 (10.4%) | 0.87 |
| Vasopressors use, n (%) † | 298 (84.6%) | 105 (91.3%) | 0.10 |
| RRT during ICU stay, n (%) | 71 (20.1%) | 38 (33.0%) | 0.006 |
| **Subtype membership** |  |  |  |
| Subtype B, n (%) ‡ | 147 (41.7%) | 76 (66.0%) | <0.001 |

Continuous variables were expressed as median (IQR) and were compared with the Mann-Whitney U test. Categorical variables were expressed as numbers (%) and were compared with the Fisher exact test or the Chi square test as appropriate.

† At inclusion.

‡ At ICU discharge.

Abbreviations: ICU, intensive care unit; BMI, body mass index; COPD, Chronic obstructive pulmonary disease; SAPS II, Simplified Acute Physiologic Score; SOFA, Sequential Organ Failure Assessment; RRT, renal replacement therapy; IQR, interquartile range.

**e-Table 7 Cox proportional hazards models to adjust for confounding (Charlson age-comorbidity index, duration of ICU stay, SAPS II on admission, renal SOFA score at ICU discharge) for one-year mortality**

|  | **Adjusted HRs** | **CI 95%** | ***P* value** |
| --- | --- | --- | --- |
| **Model with subtypes at ICU discharge**  Harrell’s C-index = 0.71 (95% CI 0.66-0.75)  Optimism < 0.01 |  |  |  |
| Subtype (A as reference) | 1.80 | (1.19-2.77) | 0.005 |
| Charlson age-comorbidity index | 1.24 | (1.15-1.34) | <0.001 |
| Duration of ICU stay (days) | 1.00 | (0.99-1.01) | 0.36 |
| SAPS II on admission (per 10-points increase) | 1.09 | (0.99-1.21) | 0.06 |
| Renal SOFA score at ICU discharge | 0.99 | (0.83-1.16) | 0.86 |

After adjustment for Charlson Age-Comorbidity Index, duration of ICU stay, SAPS II on admission and renal SOFA score at ICU discharge, membership in subtype B at ICU discharge was independently associated with one-year mortality. The model calibration was good according to the Grønnesby-Borgan test (*P* = 0.5).

Abbreviations: HR, Hazard ratio; CI 95%, 95% Confidence interval**,** ICU, intensive care unit; SAPS II, Simplified Acute Physiologic Score; SOFA, Sequential Organ Failure Assessment.

**e-Table 8 Cox proportional hazards models to adjust for confounding (age, chronic kidney disease, diabetes mellitus, duration of ICU stay, SAPS II on admission, SOFA score at ICU discharge) for one-year mortality**

|  | **Adjusted HRs** | **CI 95%** | ***P* value** |
| --- | --- | --- | --- |
| **Model with subtypes at ICU discharge**  Harrell’s C-index = 0.70 (95% CI 0.64-0.74)  Optimism < 0.01 |  |  |  |
| Subtype (A as reference) | 1.62 | (1.03-2.54) | 0.037 |
| Age (per 10-years increase) | 1.29 | (1.09-1.53) | 0.03 |
| Chronic kidney disease | 1.77 | (1.04-3.00) | 0.033 |
| Diabetes mellitus | 1.21 | (0.78-1.90) | 0.38 |
| Duration of ICU stay (days) | 1.00 | (0.99-1.01) | 0.64 |
| SAPS II on admission (per 10-points increase) | 1.09 | (0.97-1.22) | 0.13 |
| SOFA score at ICU discharge | 0.99 | (0.95-1.04) | 0.91 |

After adjustment for age, chronic kidney disease, diabetes mellitus, duration of ICU stay, SAPS II on admission and renal SOFA score at ICU discharge, membership in subtype B at ICU discharge was independently associated with one-year mortality. The model calibration was good according to the Grønnesby-Borgan test (*P* = 0.4).

Abbreviations: HR, Hazard ratio; CI 95%, 95% Confidence interval**,** ICU, intensive care unit; SAPS II, Simplified Acute Physiologic Score; SOFA, Sequential Organ Failure Assessment.

**e-Table 9 Initial and reduced biomarker regression models to discriminate the two subtypes at ICU discharge.**

|  | **Adjusted ORs** | **CI 95%** | ***P* value** |
| --- | --- | --- | --- |
| **Initial biomarker regression model**  AUC = 0.88 (95% CI 0.85-0.92)  Optimism < 0.01 |  | | |
| Hs-cTnI (per 10-μg/L increase) | 1.0 | (0.99-1.00) | 0.73 |
| Bio-ADM (per 10-pg/mL increase) | 1.09 | (1.03-1.19) | 0.008 |
| Interleukin-6 (per 10-pg/mL increase) | 1.01 | (0.98-1.03) | 0.4 |
| BNP (per 10-pg/mL increase) | 1.01 | (1.000-1.02) | 0.015 |
| Procalcitonin (per 1-ng/l increase) | 1.39 | (1.12-1.84) | 0.008 |
| Galectin 3 (per 1-ng/mL increase) | 1.60 | (1.09-2.45) | 0.02 |
| Plasma cystatin C (per 1-mg/L increase) | 4.64 | (2.41-9.52) | <0.001 |
|  |  |  |  |
| **Reduced biomarker regression model** |  | | |
| Bio-ADM (per 10-pg/mL increase) | 1.10 | (1.03-1.19) | 0.006 |
| BNP (per 10-pg/mL increase) | 1.01 | (1.0-1.02) | 0.014 |
| Procalcitonin (per 1-ng/l increase) | 1.41 | (1.13-1.86) | 0.006 |
| Galectin 3 (per 1-ng/mL increase) | 1.63 | (1.11-2.49) | 0.02 |
| Plasma cystatin C (per 1-mg/L increase) | 4.51 | (2.35-9.21) | <0.001 |

The difference in AUCs (i.e., discrimination) was not significant between the two biomarker regression models (Delong test, *P*= 0.8).

Abbreviations: ICU, intensive care unit; OR, Odds ratio; CI 95%, 95% Confidence interval**;** AUC, area under the curve; Hs-cTnI, high-sensitive cardiac troponin I; Bio-ADM, Bio-adrenomedullin; BNP, Brain natriuretic peptide.

**e-Table 10 Characteristics of the main clinical studies using an unsupervised approach (i.e., phenotyping) to identify different classes in sepsis-survivors after ICU discharge.**

| Study (year) | Design | Study population | Population size | Phenotyping method | Phenotyping variables | Follow up | Main results |
| --- | --- | --- | --- | --- | --- | --- | --- |
| Yende et al. (2019)  [11] | Post hoc study: reanalysis of an RCT evaluating a primary care-based sepsis after care intervention | Adult survivors of sepsis | *N*= 291 (complete data available on 159 participants) | Longitudinal trajectories were clustered by factor analysis | Longitudinal trajectories of Physical Component Scores of the SF-36 | 24 months after ICU discharge | Two different physical recovery trajectories were identified. Older patients with more co-morbidities and lower educational levels were more likely to have a poor physical recovery. |
| Puthucheary et al. (2020) [29] | Prospective, multicenter cohort study | Adult survivors of sepsis | *N*= 483 | Joint latent class mixture model | Latent biomarkers of inflammation and immunosuppression (i.e., hs-CRP and sPDL1*) trajectories at 6 months | 12 months after ICU discharge | Two phenotypes were identified: hyperinflammation and immunosuppression phenotype group and normal phenotype group. Compared with normal phenotype, the hyperinflammation and immunosuppression phenotype had higher 1-year mortality. |
| Soussi et al. (current study) | Post hoc study: reanalysis of a prospective observational multicenter study (FROG-ICU cohort) | Adult survivors of sepsis | *N*= 467 | Latent class analysis (i.e., mixture model) | 15 clinical and biological data available at the time of ICU discharge | 12 months after ICU discharge | Two distinct clinical subtypes were identified. Patients assigned to subtype B (47%) had more impaired cardiovascular and kidney functions, hematological disorders, and inflammation at ICU discharge than subtype A. Subtype B was independently associated with increased one-year mortality |

Abbreviations: RCT, randomized controlled trial; SF-36, short form-36 questionnaire; ICU, intensive care unit; hs-CRP, highly sensitive C-reactive protein; sPD-L1, soluble programmed death ligand 1.
